# Genomic and Immune Profiling of a Patient With Triple-Negative Breast Cancer That Progressed During Neoadjuvant Chemotherapy Plus PD-L1 Blockade

**DOI:** 10.1200/PO.18.00335

**Published:** 2019-05-10

**Authors:** David Casadevall, Xiaotong Li, Ryan L. Powles, Vikram B. Wali, Natalia Buza, Vasiliki Pelekanou, Arjun Dhawan, Julia Foldi, Borbala Szekely, Francesc Lopez-Giraldez, Christos Hatzis, Lajos Pusztai

**Affiliations:** ^1^ Yale School of Medicine, New Haven, CT; ^2^ Institut Hospital del Mar d’Investigacions Mèdiques, Barcelona, Spain; ^3^ National Institute of Oncology, Budapest, Hungary; ^4^ Yale Center for Genome Analysis, New Haven, CT

## INTRODUCTION

Preliminary results from neoadjuvant trials combining immune checkpoint blockade (ICB) with standard-of-care chemotherapy suggest high pathologic complete response rates that range between 50% and 80% in triple-negative breast cancer (TNBC).^1-3^ Adding atezolizumab to NAB-paclitaxel for first-line treatment of metastatic TNBC significantly improved response rate and progression-free survival compared with NAB-paclitaxel alone, suggesting synergy between ICB and chemotherapy.^4^ However, not all patients respond to ICB, and a minority exhibit rapid progression of their disease.^5^ Patients who experience exceptionally favorable or unfavorable responses provide unique opportunities for studying disease biology and for identifying response markers. Progression during neoadjuvant chemotherapy is a rare event in TNBC. Here, we report results from the molecular analysis of a TNBC that rapidly progressed during neoadjuvant chemotherapy plus programmed death-ligand 1 (PD-L1) blockade in a clinical trial Neoadjuvant MEDI4736 Concomitant With Weekly NAB-paclitaxel and Dose-dense AC for Stage I-III Triple Negative Breast Cancer (ClinicalTrials.gov identifier: NCT02489448). Among the first 30 patients in this ongoing trial, no other participant experienced disease progression. We also hoped to identify potentially actionable genomic alterations or immunologic features.

## CASE REPORT

The 41-year-old premenopausal woman presented with a self-palpated lump in the right breast, and mammogram revealed multifocal T1N1 disease. Core needle biopsy (CNB) of the largest breast lesion (1.7 cm) and of an enlarged right axillary lymph node showed high-grade TNBC. Systemic staging was negative for distant metastases, and germline cancer-susceptibility panel testing revealed no deleterious mutations. Baseline tumor-infiltrating lymphocyte (TIL) count was 10% (CD4, 5%; CD8, 5%; and CD20, 1%), macrophage (CD68) was 1%, and tumor cellularity was 50%. TILs were both intratumoral and stromal (ie, this was not a T-cell excluded tumor). The patient agreed to participate in a neoadjuvant phase I/II clinical trial that combined durvalumab (10 mg/kg once every 2 weeks) with once-per-week NAB-paclitaxel (100 mg/m^2^) for 12 cycles and dose-dense doxorubicin plus cyclophosphamide (ddAC) for 4 cycles. After 8 weeks of NAB-paclitaxel plus durvalumab, physical examination showed increased tumor size and new skin edema confirmed by repeat mammogram and ultrasonogram. Repeat CNB showed 60% tumor cellularity but also an increase in TIL count to 20% (CD4, 10% to 15%; CD8, 5%; and CD20, 0%) and an increase in macrophages (CD68, 20%). TILs were again noted in the stroma and intratumorally. NAB-paclitaxel was stopped but because of the increased immune infiltration, durvalumab was continued, and the patient was administered ddAC, which led to disease stabilization after four courses of therapy. Because of the apparent clinical benefit, she received two additional courses of ddAC without durvalumab off protocol and underwent right skin-sparing mastectomy and lymph node dissection. Pathology showed extensive multifocal disease (largest focus, 2.4 cm; tumor cellularity, 40%) with lymphovascular invasion in the breast and more than 10 positive axillary lymph nodes (ypT2, ypN3). Immune cell proportions in the mastectomy were TILs, 20%; CD4, 10%; CD8, 10%; CD20, 1%; and CD68, 10%.

DNA and RNA were extracted from formalin-fixed paraffin-embedded sections of tumor samples obtained at baseline, at week 8, and from the mastectomy. Blood DNA served as the reference for somatic mutation calling for whole-exome sequencing. Immune gene messenger RNA expression analysis was performed by using the Nanostring PanCancer Immune Profiling assay (Nanostring Technologies, Seattle, WA) as described in the Data Supplement and as previously reported.^6,7^ The patient’s gene expression levels were compared with those from a reference cohort (n = 31) of untreated primary breast cancers analyzed with the same platform.^6^ The patient’s result was considered to be a significant outlier if her expression levels fell outside the 2.5th to 97.5th percentile range of the bootstrap distribution of the same gene in the reference cohort. TIL counts and immune cell subtypes were determined by routine pathology and immunohistochemistry.

**FIG 1.**
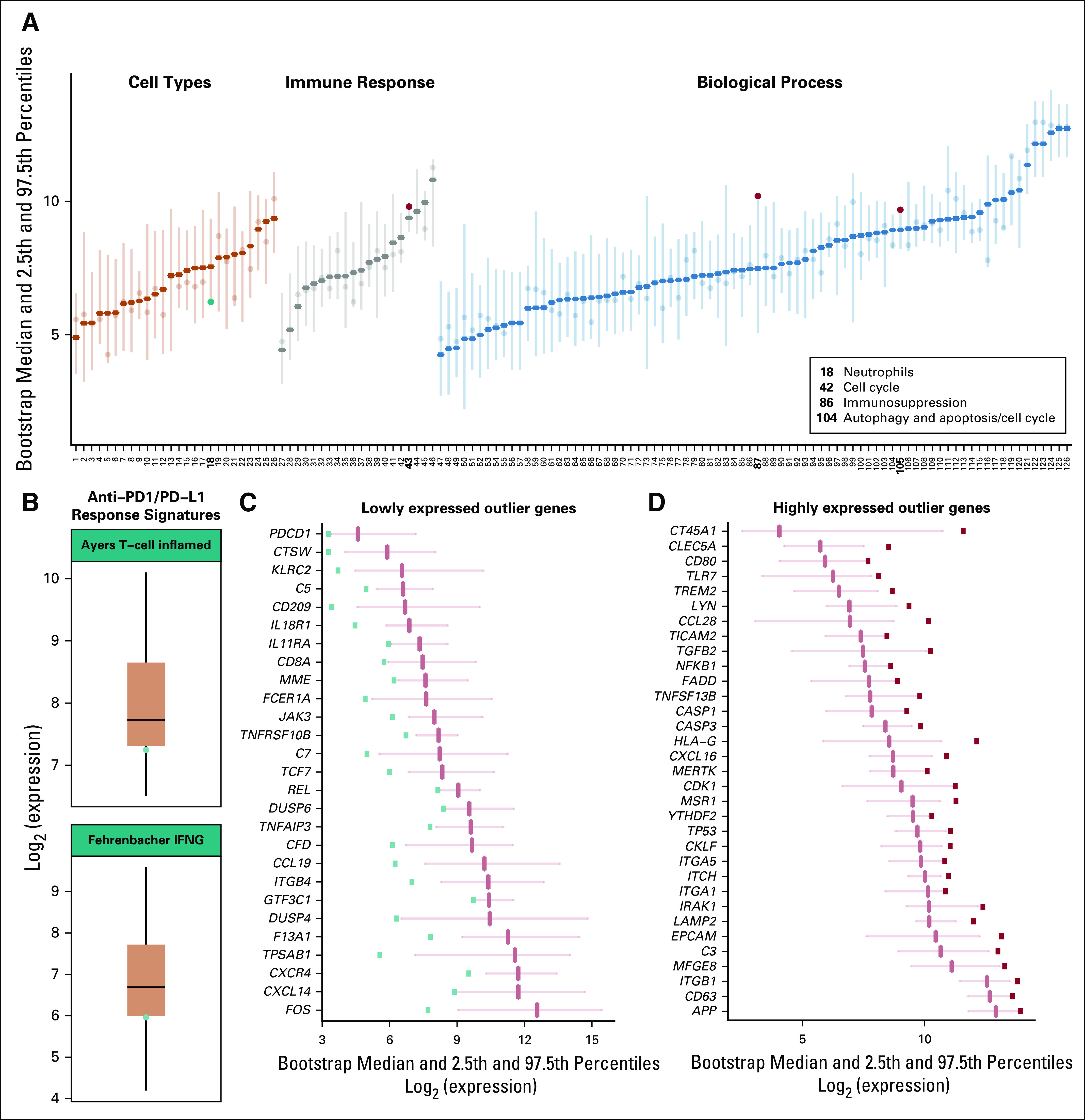
Patient’s baseline characteristics. (A) Bootstrap distribution of each category ranked by median values and category. For each category, the median reference distribution and the 2.5th to 97.5th percentile range is shown. Patient expression values for each category are shown as dots. Categories in which our patient’s score is above the 97.5th percentile (red dots) or below the 2.5th percentile (teal dots) are highlighted, are shown as bold numbers, and are described in the legend key. (B) Boxplots showing distribution of signature expression in the reference cohort. The box shows the interquartile range, and the whiskers represent 1.5× the interquartile range from the top and bottom quartiles. Teal dots show our patient’s expression value for each signature, which is in the lowest quartile for both signatures. Finally, genes for which our patient had expression values below the 2.5th (panel C, teal squares) or above the 97.5th (panel D, red squares) bootstrap distribution percentiles are shown. IFNG, interferon gamma signature.

When we compared our patient’s baseline expression of 26 immune cell types and 100 immune function metagenes with those in the reference cohort, her neutrophil metagene expression was below the 2.5th percentile, whereas the cell cycle and immunosuppression metagenes were above the 97.5th percentile of the reference distribution (Fig 1A), indicating a highly proliferative tumor with an immunosuppressed microenvironment. All other metagene categories were within the 2.5th to 97.5th percentile range (Data Supplement). The tumor also showed low expression of two previously validated immunotherapy predictive gene signatures,^8,9^ indicating low probability of response to ICB (Fig 1B). Single-gene level analysis revealed significantly low expression of 27 genes including *PDCD1*, *CD8A*, and *KLRC2* (Fig 1C), suggesting impaired T-cell and natural killer (NK) cell activity. In addition, we found high expression of 33 genes, including *TGFB*, *HLA-G*, *CD63*, *CCL28*, and *CXCL16* (Fig 1D), which are associated with an immune evasive phenotype, increased cell motility, and invasion. Baseline expression status of all genes is provided in the Data Supplement.

**FIG 2.**
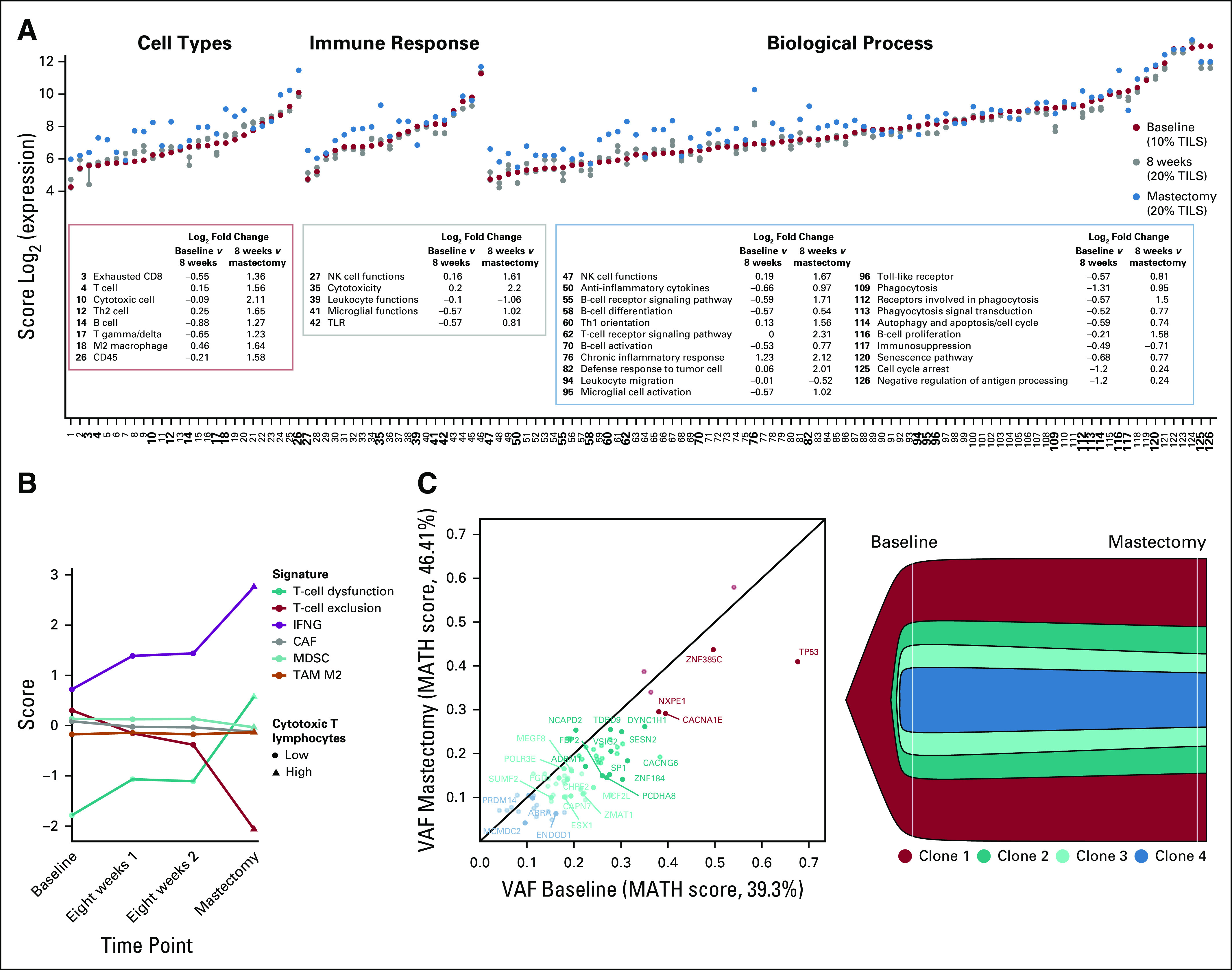
Tumor clonal evolution and dynamic changes in immune microenvironment. (A) Expression scores of each category at all three different time points are shown, ranked by baseline expression value and annotation category. Legend boxes and bold numbers show categories for which we observed an increase or decrease of ≥ 0.5 log_2_ fold change between either baseline and 8 weeks (first period) and/or 8 weeks and mastectomy (second period). (B) Tumor immune dysfunction and exclusion (TIDE)-based (Data Supplement) T-cell dysfunction and exclusion shown together with correlation coefficients of the gene expression values for our patient with cancer-associated fibroblast (CAF), myeloid-derived suppressor cell (MDSC), and M2 tumor-associated macrophages (TAM M2) signatures. (C) Variant allele frequency (VAF) plot (left) and fishplot (right) illustrating the clonal architecture and evolution (based on Sciclone [Waltham, MA] and Clonevol tools; Data Supplement) of the tumor between baseline and mastectomy time points. For optimal visualization, only mutations considered to have a high functional impact (Data Supplement) are labeled. IFNG, interferon gamma signature; MATH, mutant allele tumor heterogeneity; TIL, tumor-infiltrating lymphocyte; TLR, Toll-like receptor.

Next, we examined expression changes across the three time points. Overall, greater changes were observed between week 8 and mastectomy (ddAC treatment) than between baseline and week 8 (NAB-paclitaxel treatment). We observed upregulation of *FOS*, *ABCB1*, *KIR3DL3*, *GZMA*, and *GZMK* in the mastectomy (single-gene and metagene expression changes are provided in the Data Supplement). Most immune metagenes increased during the second period (Fig 2A), consistent with an increase of immune cell (mainly T and NK cells) infiltration. The upregulation of *ABCB1*, a multidrug resistance drug efflux pump, was also apparent and may have contributed to chemotherapy resistance in tumor cells. We also observed a concurrent decrease in immunosuppression, leukocyte functions, and leukocyte migration metagene categories, suggesting a potentially ineffective immune response. To study this further, we next analyzed T-cell exclusion and dysfunction features using the tumor immune dysfunction and exclusion (TIDE) method (Data Supplement).^10^ In concordance with our previous findings, cytotoxic T-cell infiltration increased during the second period. However, this was accompanied by an increase in T-cell dysfunction score, indicating an ineffective immune response (Fig 2B).

We also performed whole-exome sequencing of the baseline and mastectomy specimens. We identified 80 somatic mutations, including one cancer driver gene, *TP53* (R175H). Other mutations with the highest variant allele frequency at baseline included *ZNF385C*, *CACNA1E*, *NXPE1*, and *DYNC1H1*, which have poorly understood functions in cancer (Fig 2C; Data Supplement). We also detected amplifications in *FGFR1*, *FGF2*, *FGF3*, *MYC, MCL1*, *CCND1*, and *TGFB2* and deletions in *CDKN2A* and *CDKN2B* (Data Supplement). By using Sciclone (Waltham, MA)^11^ and ClonEvol,^12^ we identified four distinct tumor clones, all present at baseline and in the mastectomy specimen, with little evidence for clonal selection during therapy (Figs 2C-D). In the germline, 32 highly functional impact variants were detected by using Ingenuity Variant Analysis (QIAGEN Bioinformatics, Redwood City, CA; Data Supplement). Of note, the patient was heterozygous for an 11-nucleotide frameshift-indel + insertion in the MH2 domain of the *SMAD6* gene (NC_000015.9:g.67073715_67073726delinsA). This domain is essential for the function of *SMAD6* as a negative regulator of TGFβ signaling. SMAD6 sequesters SMAD2 in the cytoplasm and prevents its translocation to the nucleus.^13^ Another potentially relevant germline variant was a heterozygous *JAK3* frameshift mutation in the JH5 domain (NC_000019.9:g.17953278delG). JAK3 is expressed in T and NK cells and mediates signal transduction by different interleukin receptors. Compound heterozygous *JAK3* inactivating mutations cause severe combined immune deficiency.^14^

## DISCUSSION

This cancer showed primary resistance to NAB-paclitaxel and ddAC chemotherapy concurrent with an anti-PD-L1 agent. The tumor harbored several poor prognostic genomic alterations at diagnosis, including a p53 mutation and coamplification of *MYC* and *MCL1*, both of which are implicated in chemotherapy resistance.^15,16^ The expression of permeability glycoprotein (P-glycoprotein; *ABCB1*), a drug-efflux transporter that mediates taxane and anthracycline resistance in vitro, also increased during treatment.^17^ We observed a measurable increase in intratumor inflammatory response by the end of the treatment (Data Supplement), but unfortunately this did not translate into clinical antitumor activity. The cancer had a persistently high TGFβ expression, and the patient also carried a germline heterozygous deletion of the negative regulator of TGFβ signaling, SMAD6. TGFβ activation in cancers has been linked to primary chemotherapy resistance and also to immune evasion and resistance to PD-L1 blockade in multiple experimental systems.^18-21^ Furthermore, the R175H p53 mutation that this cancer harbored has been shown to render cancers insensitive to the growth inhibitory effects of TGFβ.^22^ On the basis of these findings, we hypothesize that the high expression of TGFB2 by this tumor may have contributed to its immune escape and its resistance to chemotherapy. Another notable feature of this cancer was the low level of expression of programmed cell death protein 1 (PD1) at the time of diagnosis and the high level of expression of HLA-G. Low PD-L1 expression has been consistently linked to lesser benefit from ICB, and HLA-G is a self-signal that shields placental cells from immune attack, which mediates immune tolerance in pregnancy.^23^ It is impossible to ascertain which of these mechanisms played the dominant role in mediating treatment resistance in this case, or if the multiple anomalies collectively contributed to the poor outcome. However, the findings pose at least one testable therapeutic hypothesis: TGFβ-targeting therapies (eg, galunisertib, M7824, TEW7197, LY-3200882, fresolimumab, and NIS793) may have improved the efficacy of PD-L1 blockade in this particular individual.

In summary, this case demonstrates the complexity of chemotherapy and immunotherapy resistance mechanisms and suggests that multiple different biologic processes may contribute to disease progression during treatment. We find it reassuring that previously published ICB response signatures predicted low sensitivity to PD1/PD-L1 blockade for this patient. If this is confirmed in larger cohorts, these signatures could become useful for patient selection.

## Data Availability

The following represents disclosure information provided by authors of this manuscript. All relationships are considered compensated. Relationships are self-held unless noted. I = Immediate Family Member, Inst = My Institution. Relationships may not relate to the subject matter of this manuscript. For more information about ASCO's conflict of interest policy, please refer to www.asco.org/rwc or ascopubs.org/po/author-center. **Travel, Accommodations, Expenses:** Pfizer, Roche **Employment:** Sanofi **Employment:** Bristol-Myers Squibb **Stock and Other Ownership Interests:** Delphi Diagnostics **Honoraria:** Merck, AstraZeneca/MedImmune, Pfizer, Syndax Pharmaceuticals, Almac Diagnostics, Pieris Pharmaceuticals, Genentech, Immunomedics, Eisai, Seattle Genetics/Astellas Pharma, Biotheranostics **Consulting or Advisory Role:** H3 Biomedicine, Merck, Novartis, PierianDx, Seattle Genetics, Syndax Pharmaceuticals, Athenex **Research Funding:** Merck, Genentech, Seattle Genetics, AstraZeneca No other potential conflicts of interest were reported.

## References

[1] Pusztai L Hofstatter EW Chung GG et al Durvalumab (MEDI4736) concurrent with nab-paclitaxel and dose dense doxorubicin cyclophosphamide (ddAC) as neoadjuvant therapy for triple negative breast cancer (TNBC) J Clin Oncol 36 2018 (suppl; abstr 586)

[2] Loibl S Untch M Burchardi N et al Randomized phase II neoadjuvant study (GeparNuevo) to investigate the addition of durvalumab to a taxane-anthracycline containing chemotherapy in triple negative breast cancer (TNBC) J Clin Oncol 36, 2018 (suppl; abstr 104)

[3] Nanda R Liu MC Yau C et al Pembrolizumab plus standard neoadjuvant therapy for high-risk breast cancer (BC): Results from I-SPY 2 J Clin Oncol 35 2017 (suppl; abstr 506)

[4] Schmid P Adams S Rugo HS et al Atezolizumab and nab-paclitaxel in advanced triple-negative breast cancer N Engl J Med 379 2108 2121 2018 30345906 10.1056/NEJMoa1809615

[5] Ferrara R Mezquita L Texier M et al Hyperprogressive disease in patients with advanced non-small cell lung cancer treated with PD-1/PD-L1 inhibitors or with single-agent chemotherapy JAMA Oncol 4 1543 1552 2018 30193240 10.1001/jamaoncol.2018.3676 PMC6248085

[6] Szekely B Bossuyt V Li X et al Immunological differences between primary and metastatic breast cancer Ann Oncol 29 2232 2239 2018 30203045 10.1093/annonc/mdy399

[7] Shi W Jiang T Nuciforo P et al Pathway level alterations rather than mutations in single genes predict response to HER2-targeted therapies in the neo-ALTTO trial Ann Oncol 28 128 135 2017 28177460 10.1093/annonc/mdw434 PMC5834036

[8] Ayers M Lunceford J Nebozhyn M et al IFN-γ-related mRNA profile predicts clinical response to PD-1 blockade J Clin Invest 127 2930 2940 2017 28650338 10.1172/JCI91190 PMC5531419

[9] Fehrenbacher L Spira A Ballinger M et al Atezolizumab versus docetaxel for patients with previously treated non-small-cell lung cancer (POPLAR): A multicentre, open-label, phase 2 randomised controlled trial Lancet 387 1837 1846 2016 26970723 10.1016/S0140-6736(16)00587-0

[10] Jiang P Gu S Pan D et al Signatures of T cell dysfunction and exclusion predict cancer immunotherapy response Nat Med 24 1550 1558 2018 30127393 10.1038/s41591-018-0136-1 PMC6487502

[11] Miller CA White BS Dees ND et al SciClone: Inferring clonal architecture and tracking the spatial and temporal patterns of tumor evolution PLoS Comput Biol 10 e1003665 2014 25102416 10.1371/journal.pcbi.1003665 PMC4125065

[12] Dang HX White BS Foltz SM et al ClonEvol: Clonal ordering and visualization in cancer sequencing Ann Oncol 28 3076 3082 2017 28950321 10.1093/annonc/mdx517 PMC5834020

[13] Imamura T Takase M Nishihara A et al Smad6 inhibits signalling by the TGF-beta superfamily Nature 389 622 626 1997 9335505 10.1038/39355

[14] Candotti F Oakes SA Johnston JA et al Structural and functional basis for JAK3-deficient severe combined immunodeficiency Blood 90 3996 4003 1997 9354668

[15] Lee K Giltnane JM Balko JM et al MYC and MCL1 cooperatively promote chemotherapy-resistant breast cancer stem cells via regulation of mitochondrial oxidative phosphorylation Cell Metab 26 633 647.e7 2017 28978427 10.1016/j.cmet.2017.09.009 PMC5650077

[16] Gasca J Flores ML Giráldez S et al Loss of FBXW7 and accumulation of MCL1 and PLK1 promote paclitaxel resistance in breast cancer Oncotarget 7 52751 52765 2016 27409838 10.18632/oncotarget.10481 PMC5288146

[17] Yamagishi T Sahni S Sharp DM et al P-glycoprotein mediates drug resistance via a novel mechanism involving lysosomal sequestration J Biol Chem 288 31761 31771 2013 24062304 10.1074/jbc.M113.514091 PMC3814770

[18] Tauriello DVF Palomo-Ponce S Stork D et al TGFβ drives immune evasion in genetically reconstituted colon cancer metastasis Nature 554 538 543 2018 29443964 10.1038/nature25492

[19] Mariathasan S Turley SJ Nickles D et al TGFβ attenuates tumour response to PD-L1 blockade by contributing to exclusion of T cells Nature 554 544 558 2018 29443960 10.1038/nature25501 PMC6028240

[20] Flavell RA Sanjabi S Wrzesinski SH et al The polarization of immune cells in the tumour environment by TGFbeta Nat Rev Immunol 10 554 567 2010 20616810 10.1038/nri2808 PMC3885992

[21] Massagué J TGFbeta in cancer Cell 134 215 230 2008 18662538 10.1016/j.cell.2008.07.001 PMC3512574

[22] Kawarada Y Inoue Y Kawasaki F et al TGF-β induces p53/Smads complex formation in the PAI-1 promoter to activate transcription Sci Rep 6 35483 2016 27759037 10.1038/srep35483 PMC5069723

[23] Loumagne L Baudhuin J Favier B et al In vivo evidence that secretion of HLA-G by immunogenic tumor cells allows their evasion from immunosurveillance Int J Cancer 135 2107 2117 2014 24623585 10.1002/ijc.28845

